# Hox-PBC Partnership Demystified

**DOI:** 10.1371/journal.pbio.1001349

**Published:** 2012-06-26

**Authors:** Caitlin Sedwick

**Affiliations:** Freelance Science Writer, San Diego, California, United States of America

**Figure:**
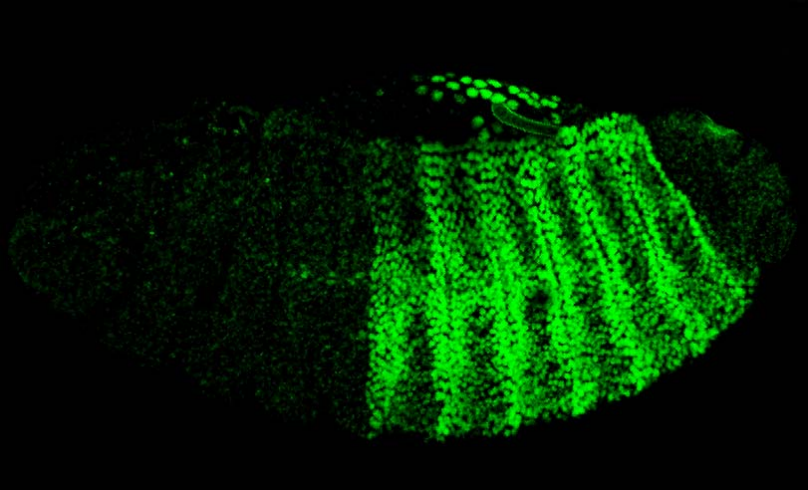
Bimolecular Fluorescence Complementation (BiFC) in action: a living stage-14 *Drosophila* embryo expressing both the Hox protein AbdA fused to one half of the fluorescent protein Venus and the PBC protein Exd fused to the other half. The green fluorescence shows where in vivo interactions between AbdA and Exd bring the two halves of Venus together to reconstitute the intact fluorescent protein.

One of the earliest steps in animal embryogenesis involves establishing the identity of embryonic structures and tissues from head to tail along the anterior-posterior axis. Hox genes encode a family of proteins that have a prominent and broadly conserved role in laying out this developmental roadmap in most animals.

For some time, it's been felt that Hox function was fairly well understood: a short amino acid sequence in Hox proteins called the hexapeptide (HX) motif drives the formation of a complex between Hox and a protein from a second family called PBC. These Hox/PBC complexes bind to DNA to regulate the expression of myriad other genes, with various combinations of Hox/PBC proteins exerting different effects on gene expression in different areas of the embryo. However, as Bruno Hudry, Samir Merabet, and colleagues show in this issue of *PLoS Biology*, the HX motif isn't actually required by all Hox proteins for the formation of Hox/PBC complexes—instead, alternative modes of interaction are the rule.

The HX motif is clearly required for proper Hox function in vitro, because earlier studies have shown that when it's mutated, Hox proteins fail to form complexes with PBC. However, while these findings suggested that Hox proteins with mutated HX motifs should impair embryonic development, recent studies failed to corroborate this expectation. This discrepancy is what motivated Hudry and colleagues to conduct a systematic survey of HX motif importance for the formation of functional Hox protein complexes in multiple species.

To test the importance of the HX motif, Hudry et al. used an approach called Bimolecular Fluorescence Complementation (BiFC). In BiFC, a fluorescent protein such as GFP is split into two pieces that are then tacked onto two other proteins whose interaction is being tested. If those two proteins bind to each other, then the fluorescent protein fragments will rejoin, allowing fluorescence to be observed. Accordingly, Hudry and colleagues fused one half of a fluorescent protein to each Hox protein they wanted to test, the other half to the PBC partner, and expressed both fusion proteins in cells or embryos. Then they looked to see whether fluorescence—indicative of interaction between the Hox and PBC proteins—could be observed.

As expected, wild-type Hox proteins bound to PBC in vivo to produce fluorescence. However, whereas the earlier, in vitro studies had suggested that mutations in the HX motif should abolish Hox/PBC interactions (and therefore fluorescence), the authors instead found that fluorescence was intact for the most of HX-mutated Hox proteins they tested. This was true whether the proteins tested came from flies or mice, and it suggested that alternative, HX-independent modes of interaction exist when Hox proteins are expressed in cells, rather than when tested in vitro.

What could account for this observation? Hudry and colleagues wondered whether some co-factor that's present in cells, but had not been present in the in vitro studies, might promote HX-independent Hox/PBC interactions. Their suspicions immediately fell upon Meis, a protein already known to participate in a three-component complex with Hox and PBC. Meis is thought to modulate Hox genes' regulatory functions, and versions of Meis have been evolutionary conserved throughout the animal kingdom, making it a likely culprit. The researchers reasoned that Meis proteins present in cells and embryos (but absent from the in vitro experiments conducted thus far) might be able to facilitate Hox/PBC complex formation when the HX motif is mutated.

The obvious next step, therefore, was to test the ability of Meis to promote HX-independent Hox/PBC complex formation, so the authors returned to the in vitro setting, using purified Hox, PBC, and Meis proteins. Their experiments showed that, in the absence of Meis, most of the Hox proteins tested required intact HX motifs in order to form complexes with PBC in vitro. However, when Meis was present, it turned out that the HX motif was dispensable (three-part complexes containing Hox, PBC, and Meis could form even when the Hox proteins' HX motifs were mutated). Only a few of the Hox proteins the authors tested showed an absolute requirement for the HX motif.

The ability of Meis to promote HX-independent complex formation appears to be highly conserved; the authors observed this activity when they compared equivalent Hox proteins in flies, mice, and even sea anemone. Furthermore, the authors found that some Hox proteins require neither Meis nor the HX motif to form complexes with PBC, instead relying on other domains within the Hox protein. Collectively, these results resolve the earlier, apparently contradictory findings on the HX motif's importance, and provide a new perspective from which to view future results.

**Hudry B, Remacle S, Delfini M-C, Rezsohazy R, Graba Y, et al. (2012) Hox Proteins Display a Common and Ancestral Ability to Diversify Their Interaction Mode with the PBC Class Cofactors. doi:10.1371/journal. pbio.1001351**

